# A Rare Case of Spindle Cell Hemangioma in the Palmar Region

**DOI:** 10.7759/cureus.48524

**Published:** 2023-11-08

**Authors:** Takahiro Yamazaki, Yusuke Matsuura, Seiji Ohtori

**Affiliations:** 1 Orthopedic Surgery, Chiba University, Chiba, JPN; 2 Orthopedics, Chiba University Hospital, Chiba, JPN; 3 Orthopedic Surgery, Graduate School of Medicine, Chiba University, Chiba, JPN

**Keywords:** recurrence, hand, palm, sch, spindle cell hemangioma

## Abstract

Spindle cell hemangioma (SCH) is an exceptionally uncommon vascular neoplasm, primarily manifesting on the extremities. This article delves into a singular case of a 20-year-old female presenting with erythema and discomfort in the left palm, diagnosed with SCH. Post-consultation with a dermatologic neoplasm consortium, she underwent a comprehensive excision, followed by a bi-phasic skin graft. Despite non-clear surgical perimeters, the patient, five years post-procedure, has experienced neither re-emergence nor functional encumbrance. The discussion emphasizes the notable recurrence propensity of SCH, with a historical recurrence rate surpassing 50%, and underscores the importance of an interdisciplinary strategy, enduring surveillance, and bespoke therapeutic decisions for management. The paper concludes by advocating for augmented research and expansive case compilations to enhance therapeutic paradigms for such atypical vascular lesions.

## Introduction

Spindle cell hemangioma (SCH) represents a very rare type of vascular tumor. It was initially described by Weiss and Enzinger in 1986, who presented the first 26 cases [1]. They concluded that this vascular lesion was a low-grade vascular tumor, thus naming it spindle cell endothelioma. Subsequent investigations, including immunohistochemical studies, established its benign nature [2]. These lesions affect both genders equally and tend to favor the extremities. There are approximately 186 cases of SCH reported in the British literature, with 40 involving the hand [3]. SCH can present as solitary (SCH) or multifocal lesions (spindle cell hemangiomatosis) [4]. Both types have been discovered in the hand. These lesions tend to manifest in childhood or early adulthood. While they mostly present as painless rough nodules [5], they can sometimes be painful and unsightly [6]. Histologically, they consist of spindle-shaped cells that do not stain with endothelial immunohistochemistry. The standard treatment involves wide local excision, but care is needed when multifocal disease is present to avoid excessive morbidity. Furthermore, the recurrence rate exceeds 50%, making treatment challenging at times [4].

## Case presentation

**Figure 1:**
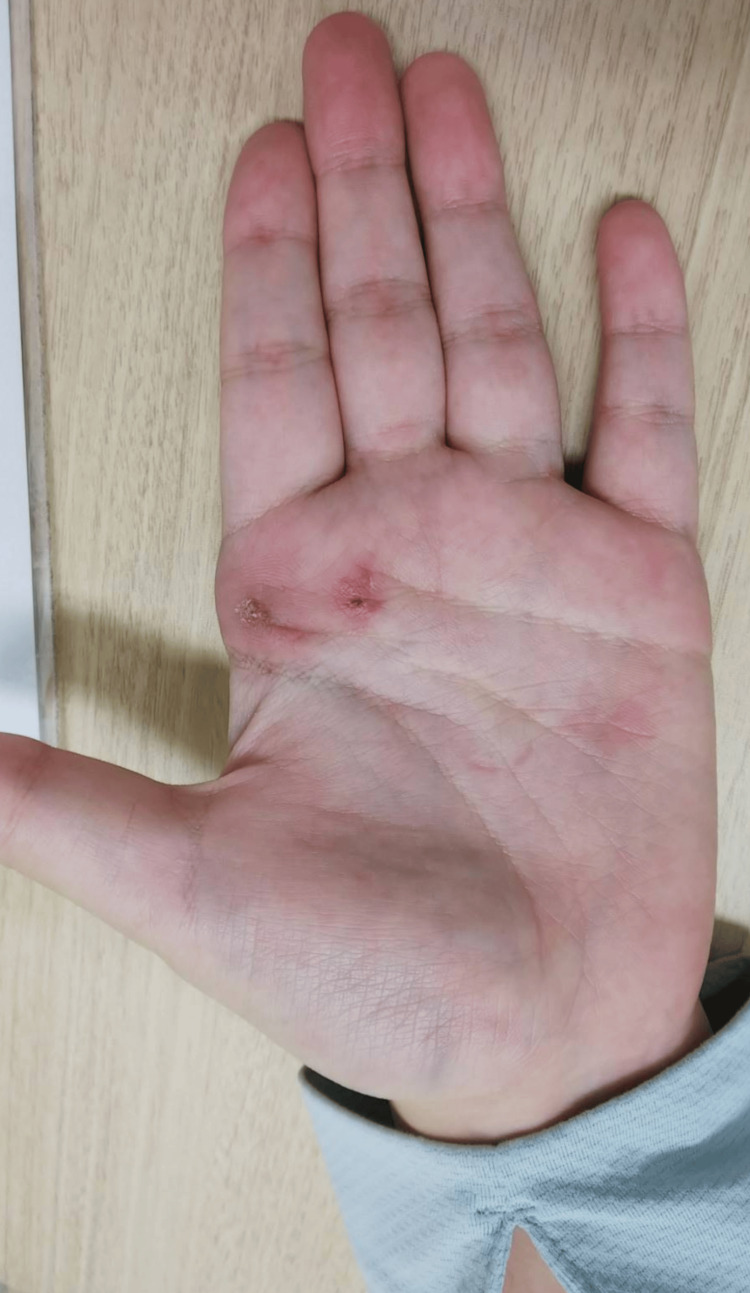
Photograph showing the appearance of the hand Prominent erythema was apparent on the palm, and the scar on the palm is the result of a biopsy.

The patient was a 20-year-old female who had noticed redness in the palmar region for several years (Figure 1). Due to the onset of pain, she sought medical attention and was referred to our dermatology department. A biopsy diagnosed her with SCH. Given the hand tumor, our department was tasked with surgery. She had palmar redness and pain, especially when gripping objects. There was no restriction in finger mobility and no similar findings in other regions.

**Figure 2:**
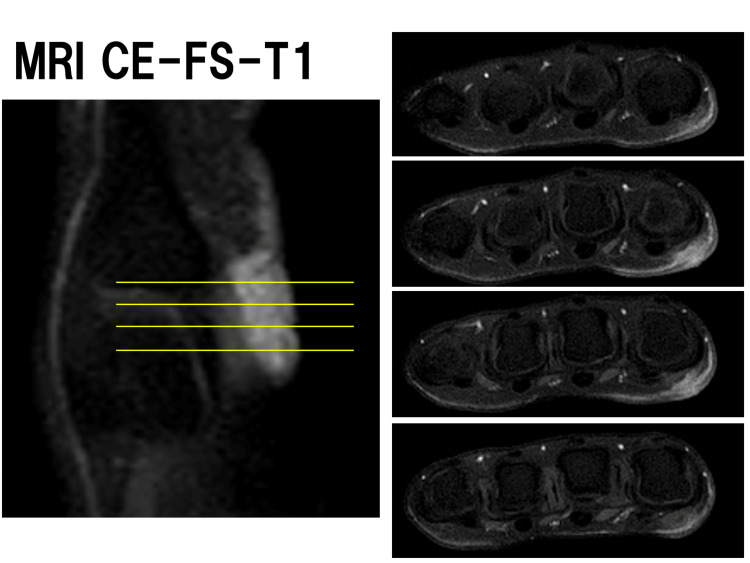
Contrast-enhanced MRI with fat-suppressed T1-weighted images MRI images showed a high-signal area at the index finger MP joint, and the axial view allowed visualization of its extent.

MRI with fat-suppressed T1-weighted images showed a high-signal area at the index finger MP joint, and the axial view allowed visualization of its extent (Figure 2).

**Figure 3:**
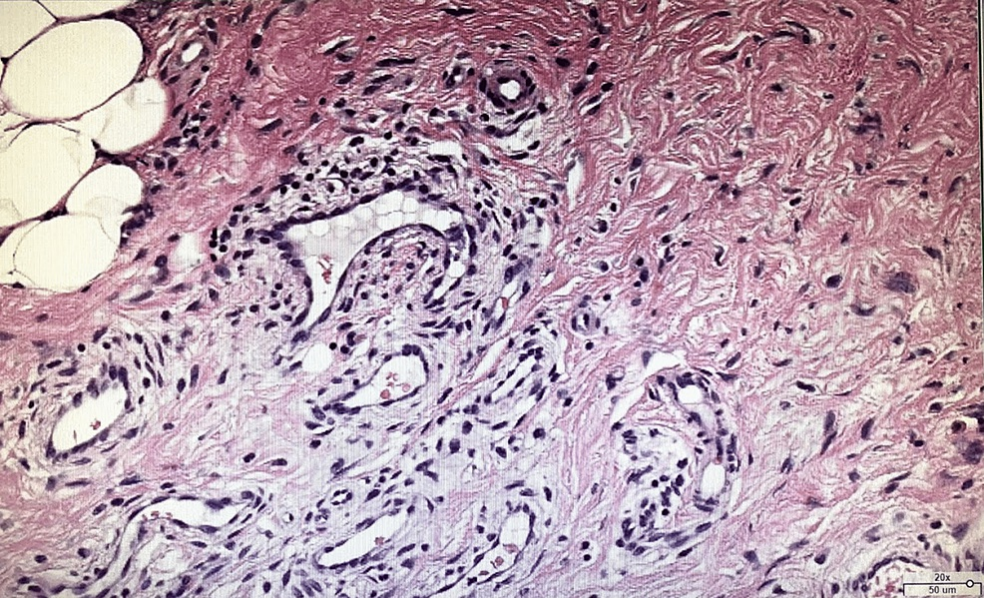
Hematoxylin and eosin staining Cellular proliferation of spindle morphology was evident.

**Figure 4:**
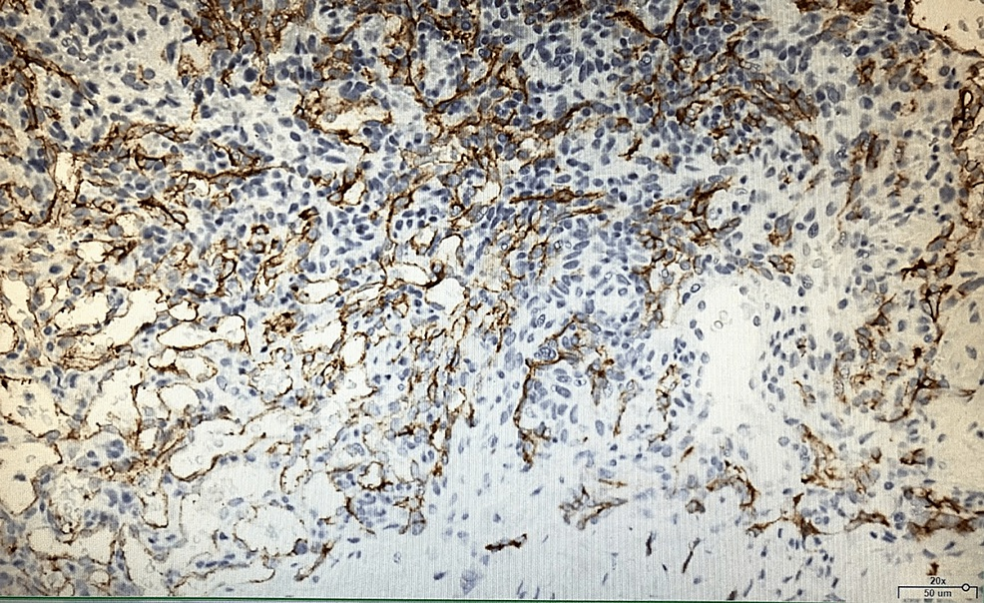
CD34/CD31 immunohistochemical staining These cells were not stained by immunostaining.

Histologically, there was an increase in spindle-shaped cells, which did not stain for CD34/CD31 (Figures 3, 4).

**Figure 5:**
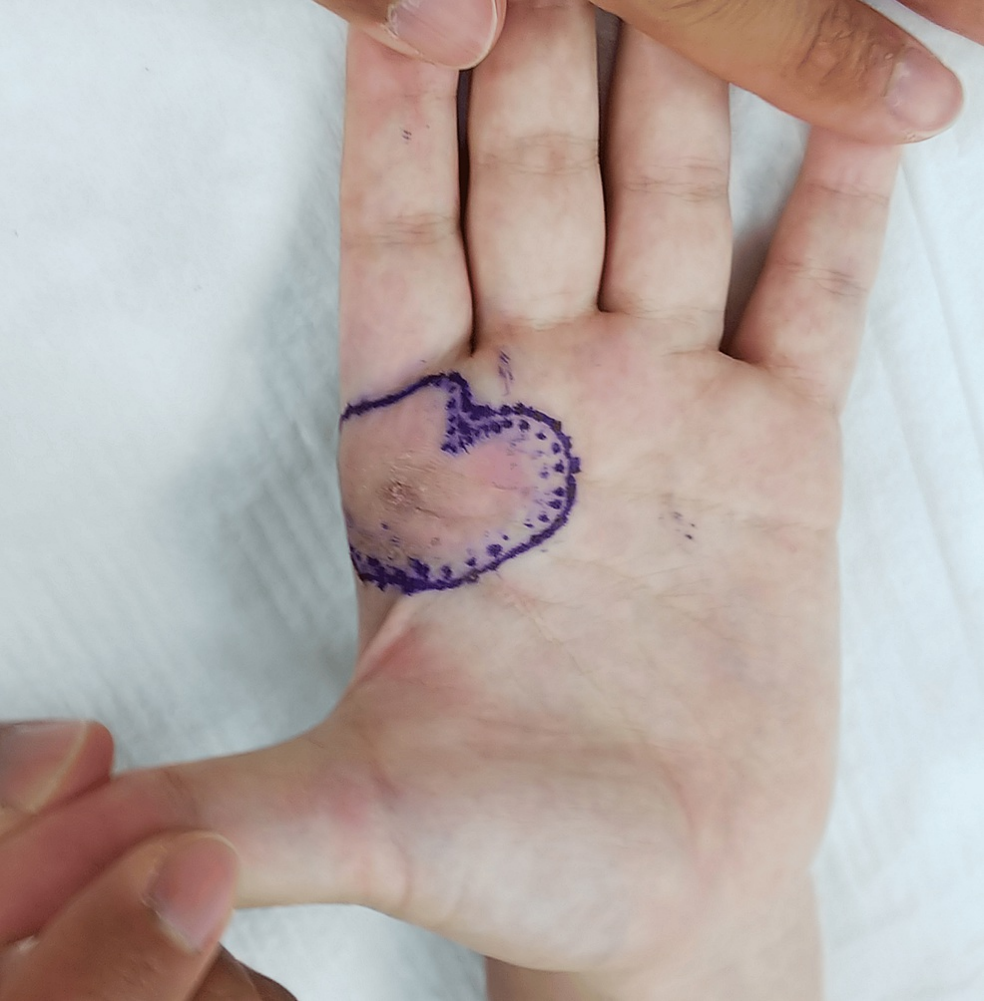
Photograph showing the boundaries of the excision We took a margin of about 3 mm from the reddened skin.

**Figure 6:**
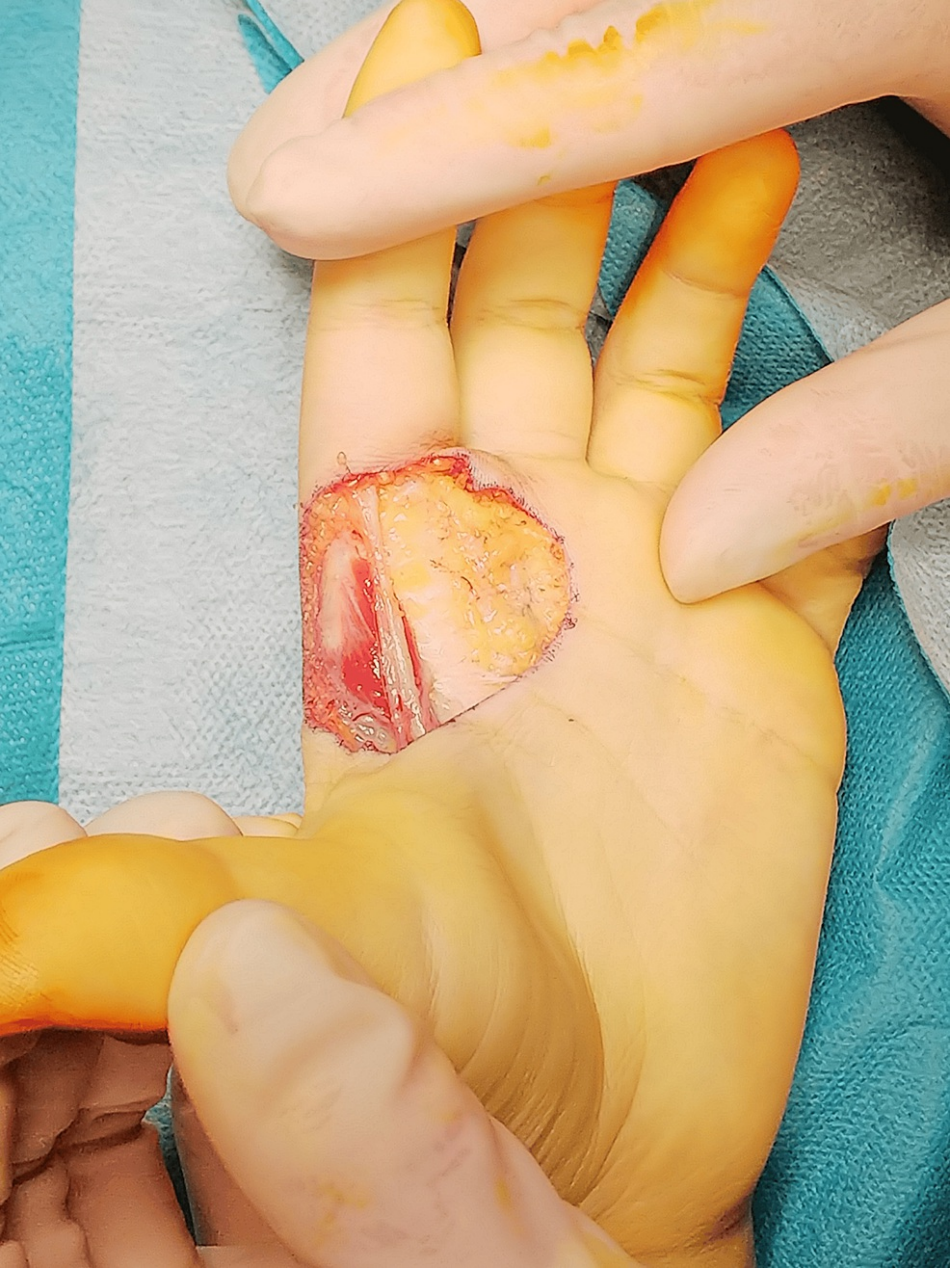
Photograph showing the palm after a wide excision

The decision was made to perform wide local excision of the soft tissue tumor after consulting with the dermatology tumor team for margin determination (Figure 5). During the first surgery, the tumor was adherent to the digital nerve but could be excised en bloc (Figure 6). A part of the nerve was in contact with the tumor, and its superficial membranous tissue was removed. The defect was covered with artificial dermis and negative pressure wound therapy (NPWT).

**Figure 7:**
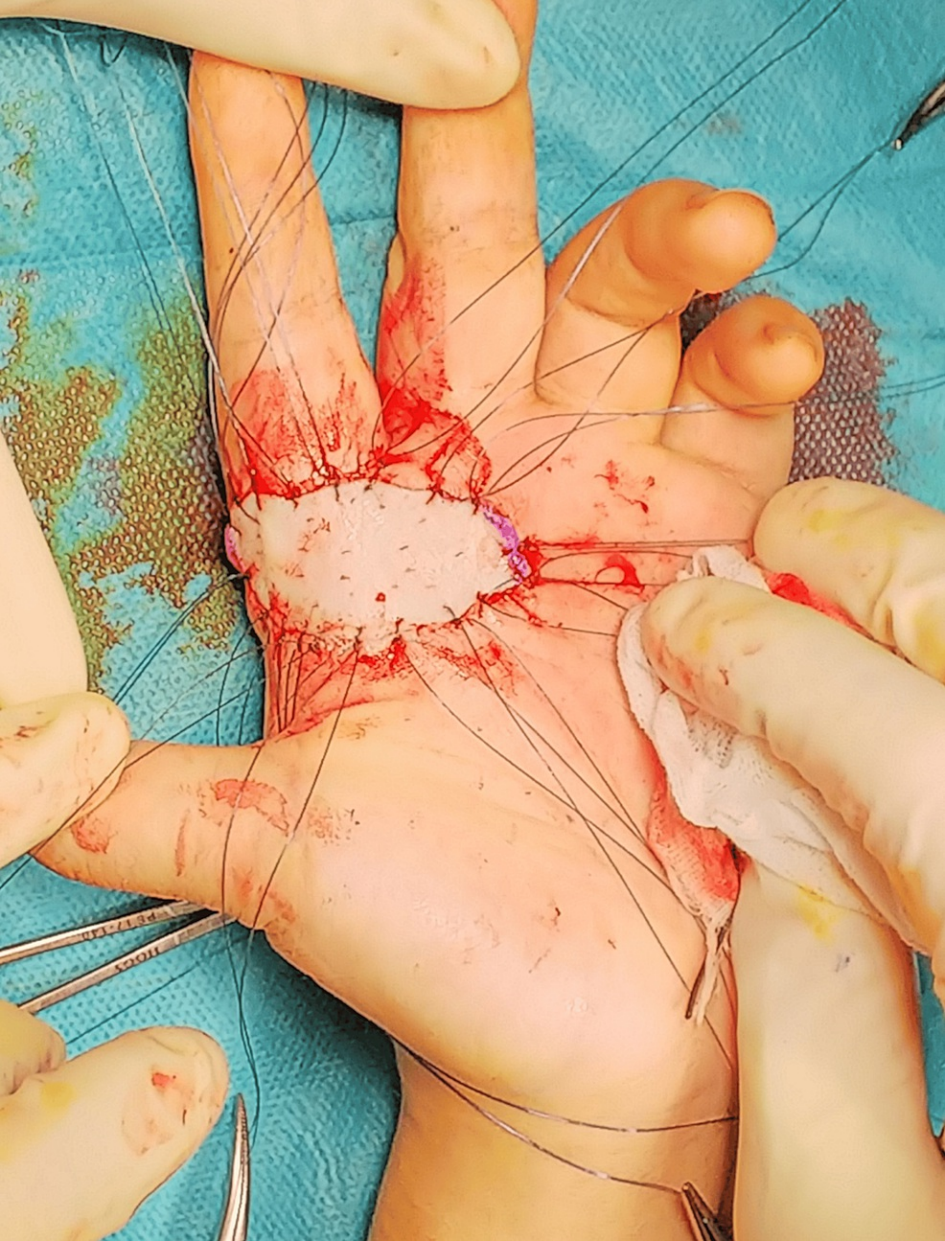
Photograph showing the palm after a skin graft We performed a full-thickness skin graft.

Although pathology results indicated positive margins, the area was near the nerve. After consultation with the patient and her family, no additional excision was done, opting instead for skin reconstruction (Figure 7). Two weeks post-surgery, skin grafting was performed using a graft from the sole of the foot to cover the palmar defect.

**Figure 8:**
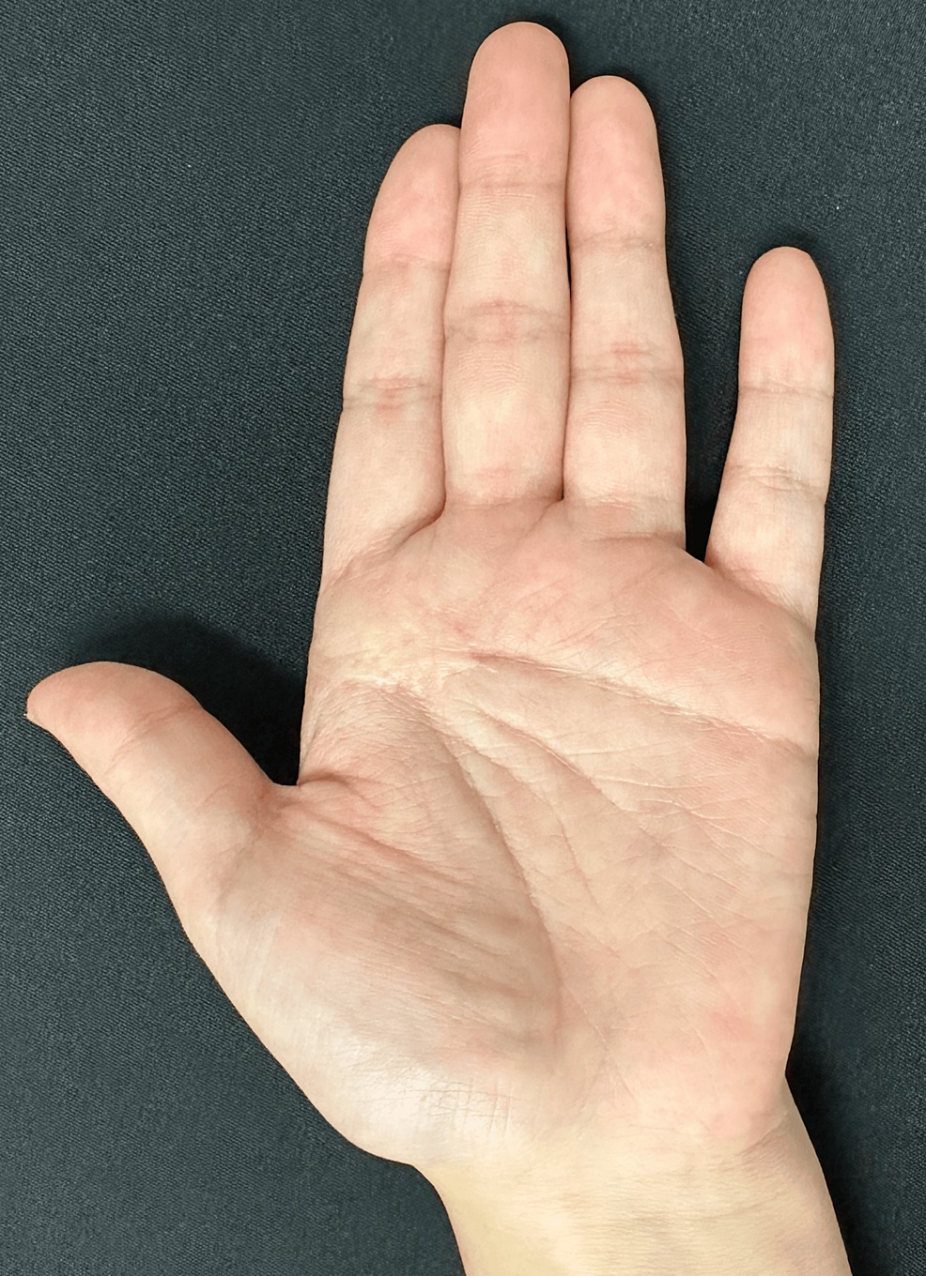
Appearance of the hand five years post-surgery There has been no recurrence, and the outcome has been good.

Five years post-operatively, the patient shows no signs of recurrence, pain, or restriction in finger mobility (Figure 8).

## Discussion

SCH has been reported in roughly 200 cases, with about 20% involving the hand. Although it is primarily a benign tumor, there are suggestions that multifocal symptomatic cases can become malignant. The recurrence rate is greater than 50%, with wide excision being recommended [3,4]. Harrison et al. reported on a 22-year-old male who developed a tumor on his entire ring finger following blunt trauma [7]. Despite embolization, there was no improvement, leading to a ray amputation [7]. Gray et al. described a case where a hand SCH recurred 14 years after simple excision [3]. After wide excision, the patient had a good postoperative course with no recurrence [3]. Perkins and Weiss noted that 58% of patients experienced “local recurrence,” all within the same general body area, from months to 34 years after the initial surgery [4]. Over a third of those patients experienced multiple recurrences. However, even among patients with recurrence, death from the lesion was rare [4].

In the present case, given its symptomatic nature and the patient’s family’s surgical preferences, wide excision was chosen to prevent recurrence. The margins were positive, suggesting that digital nerve excision might have been necessary. However, perhaps due to the removal of the nerve’s superficial membranous tissue, there was no recurrence or functional impairment even five years post-surgery. This was an instance in which the preservation of the digital nerve was successful. Continuous long-term follow-up will be necessary.

## Conclusions

SCH in the hand is a rare benign tumor with a high recurrence rate. Treatment typically involves wide excision. In this case of a 20-year-old female, surgical intervention successfully prevented recurrence for five years post-operatively, with nerve preservation. Continuous long-term monitoring remains crucial.
